# Nanovesicles for Sensitive Skin Care Developed via Self‐Assembly of Glutamine Linoleate

**DOI:** 10.1111/jocd.70195

**Published:** 2025-04-21

**Authors:** Koo Chul Kwon, Mi Jung Kim, Sang A. Yoon

**Affiliations:** ^1^ SEORIN COMPANY Co., Roundlab R&D Center Seoul Republic of Korea

**Keywords:** nanovesicles, QLAsome, self‐assembly, skin sensitivity, soothing effect

## Abstract

**Background:**

_L‐_glutamine and linoleic acid (LA) can suppress inflammatory cytokine expression; however, studies on their simultaneous application are limited due to polarity differences.

**Aims:**

To investigate the effect of glutamine linoleate vesicles (QLAsomes) on skin sensitization by assessing their impact on sensitization‐related protein expression, bacterial growth, and clinical efficacy in relieving skin itchiness.

**Methods:**

After synthesizing and analyzing QLAsomes, their inhibitory effects on capsaicin‐induced cytokine expression and *Staphylococcus aureus* growth were evaluated. In a double‐blind clinical trial, 24 participants (ages 22–63) with sensitized skin applied 10 wt% QLAsome cream on one side and a vehicle or no cream on the other twice daily for 2 weeks. Itchiness in the elbow area was assessed using a visual analog scale and expert evaluation. Skin barrier changes were measured using transepidermal water loss (TEWL), skin erythema, and stratum corneum (SC) hydration.

**Results:**

QLAsomes, formed by _L‐_glutamine and LA through hydrogen bonding, were spherical vesicles (164.6 ± 3.1 nm). Based on the inhibitory effects of _L‐_glutamine and LA on inflammation‐related factors, QLAsomes inhibited the capsaicin‐induced expression of these factors more effectively than the individual components. IL‐4 inhibition was improved by over 26%. Matrix metalloproteinase‐1, which degrades collagen, showed 32% and 23% improvements compared to _L‐_glutamine and LA, respectively. In a clinical evaluation, 10 wt% QLAsome cream reduced itching by 45% compared to before application, which is a 67% improvement compared to placebo. Skin evaluations revealed improvements in erythema (12%), TEWL (15%), and SC hydration (19%), suggesting that QLAsomes enhance the skin barrier function.

**Conclusions:**

QLAsomes showed up to 32% higher expression inhibition of key skin sensitization‐related factors than individual components, and based on this, improved pruritus by 67% more than placebo. As nanovesicles with skin‐soothing properties, they are effective for drug encapsulation and managing skin sensitivity in pharmaceutical and cosmetic industries.

## 1 Introduction

Skin sensitivity is a clinical condition characterized by discomfort and abnormal skin sensations including tightness, stinging, burning, tingling, pain, and itching. Sensitive skin refers to a hypersensitive skin condition resulting from a weakened skin barrier, nerve sensation abnormalities, and other factors that can deteriorate the skin barrier or contribute to skin diseases such as roseola, psoriasis, and atopic dermatitis (AD) [1, 2]. If skin sensitivity persists or worsens, it can lead to sensitive skin, increasing the risk of various skin diseases. Sensitivity symptoms are primarily triggered by UV radiation, heat, and air pollutants; however, psychological and hormonal factors have also been implicated [3]. Approximately 50% of individuals worldwide (60% women and 40% men) experience skin sensitivity, with the associated discomfort significantly impacting quality of life.

However, the pathophysiological mechanisms underlying skin sensitivity remain unclear. Transient receptor potential vanilloid 1 (TRPV1), a calcium‐permeable transient receptor potential channel, plays an important role. TRPV1 is activated by endogenous inflammatory mediators and exogenous activators such as capsaicin, triggering inflammatory responses. It is widely expressed in skin tissues, including keratinocytes, peripheral sensory nerve fibers, and immune cells [4, 5]. Inflammatory cytokine activation induces skin sensitization, weakens the skin barrier, and transmits itch signals from sensitized skin. This can exacerbate stress, anxiety, and mood disorders. Persistent itchiness leads to continuous scratching, further compromising the quality of life [6].

Plant‐derived ingredients can modulate the production and activity of various inflammatory mediators, such as arachidonic acid metabolites, peptides, and key inflammatory molecules, including inducible nitric oxide synthase, cyclooxygenase, cytokines, and proteases [7]. Many plant‐based raw materials have garnered popularity in the cosmetic industry because of the growing demand for safe and clean beauty products. Black soybean ( *Glycine max* (L.) Merr.) is known for its immune and analgesic properties, containing a rich composition of amino acids, fatty acids, isoflavones, anthocyanins, and phytoestrogens such as genistein [8, 9]. However, for effective skin sensitization applications, it is necessary to consider the high efficacy of the component as well as factors such as the ease of extraction due to its rich content and suitability for cosmetic or pharmaceutical formulations. Amino and fatty acids are notable components, as they are the most abundant in black beans and can be easily and effectively applied.

_L‐_glutamine is the most abundant amino acid in black soybeans, and its topical use for alleviating skin conditions has increased in recent years [10]. It plays a role in gene expression regulation, protein synthesis, immune function, cell proliferation, anti‐inflammatory and antioxidant activity, and the suppression of allergic contact dermatitis and late anaphylaxis, a systemic hypersensitivity allergic disease [11, 12, 13, 14] Soybeans are rich in linoleic acid (LA), which comprises 57% of its total fatty acids. LA, a major component of ceramides, aids skin barrier recovery and reduces inflammation by suppressing cytokines. LA deficiency is associated with hair loss and impaired wound healing [15].

*Staphylococcus aureus* , a common skin flora constituent, is present in 2%–25% of normal skin but increases to 78%–100% in patients with AD. Its toxins act as superantigens, playing a crucial role in AD induction and aggravation by directly activating sensory neurons, causing pruritus [16]. LA alleviates pruritus and AD by suppressing cytokines and exerting antibacterial effects on *S. aureus* [17]. However, owing to differences in polarity, methods for evaluating the effects of simultaneously applied _L‐_glutamine and LA are limited. Some studies have explored effective LA delivery via π–π complexation of carboxylic groups. However, studies examining supramolecular complex formation with water‐soluble agents (e.g., amino acids) via hydrogen bonding are lacking. Additionally, studies on the effects of simultaneous application of LA and _L‐_glutamine on skin improvement are also lacking [18].

In this study, we aimed to determine the soothing effects of nanovesicles formed by ion pairing of _L‐_glutamine and LA (we named, “QLAsomes”) on skin sensitivity. Previous studies suggested that ion pairing enhances solubility and improves skin permeability. These techniques have been used to dissolve poorly soluble agents and enhance transdermal delivery, thereby improving skin health [19, 20]. After confirming the binding characteristics and morphological features of spherical nanovesicles of QLAsomes, we evaluated their suppressive effects on inflammatory cytokines induced by capsaicin and their antibacterial activity against *S. aureus* , which contributes to skin sensitization and AD.

We specifically investigated how the simultaneous application of _L‐_glutamine and LA affected the suppression of capsaicin‐induced inflammatory cytokines. In addition, a clinical trial was conducted to assess the immediate and preventive soothing effects of QLAsomes in participants with sensitive skin. We evaluated sensory improvements, skin barrier enhancement, and changes in erythema and hydration following QLAsome application, highlighting its potential as an effective cosmetic ingredient for the management of sensitive skin.

## 2 Materials and Methods

### 2.1 Formation and Analysis of QLAsome

_L_‐glutamine (Sigma‐Aldrich Inc., St. Louis, MO, USA) was dissolved in deionized (DI) Milli‐Q water by stirring at 60°C and 1400 rpm. LA (Sigma‐Aldrich Inc., St. Louis, MO, USA) was dispersed in 1,2‐hexanediol (Sigma‐Aldrich Inc., St. Louis, MO, USA) and slowly added to an aqueous _L‐_glutamine solution. This solution was mixed using a homomixer at 60°C for 20 min until a uniform and transparent liquid without any precipitate was obtained. It was manufactured with a molar ratio of _L‐_glutamine and LA of 1:1. No further purification was carried out. Macroscopic stability was assessed at room temperature, and the cells were observed for 3 months. Quercetin‐loaded QLAsomes were prepared similarly. Briefly, 0.10 g of quercetin was dispersed in ethyl alcohol (2.5 g) at 60°C. Dipropylene glycol (1.5 g) was slowly added during the mixing process using a homomixer and mixed for 20 min. No additional purification steps are required.

^1^H‐nuclear magnetic resonance (NMR) analysis was performed to validate the interbonding between _L‐_glutamine and LA. Milli‐Q water was replaced with deuterium oxide (99 at.% D_2_O; Sigma‐Aldrich) during the QLA‐some manufacturing process. Dimethyl sulfoxide‐d6 (99 atom % D, Sigma‐Aldrich) was used for structural analysis of LA and 1,2‐hexanediol. The ^1^H‐NMR spectra were measured using an AvanceIII‐500 (NMR spectrometer, Bruker Instruments, Billerica, MA, USA) operating at 500 MHz in D_2_O solution at 35°C. Tetramethylsilane was used as an internal standard. The changes in the functional groups were analyzed by Fourier transform infrared spectroscopy (FTIR) using Vertex70 and Hyperion 2000 (FTIR spectrophotometer, Bruker Instruments) over a spectral range of 4000–400 cm^−1^ at a data acquisition rate of 1.0 cm^−1^ per point. The spectra were developed and deconvoluted using the Opus 5.5 software (Bruker Instruments). The thermal properties were measured using Mettler‐Toledo DSC (Mettler‐Toledo, Columbus, OH, USA). Endothermic heat flow and temperature were recorded with accuracies of 0.00010 K and 0.010 K, respectively. The measurements were performed under a purified nitrogen atmosphere at flow and heating rates of 20 cc min^−1^ and 10 K min^−1^, respectively. The morphology of QLAsomes and quercetin‐loaded QLAsomes was analyzed by transmission electron microscopy (TEM) using a JEM‐2010 instrument (Jeol Ltd., Tokyo, Japan). The samples were stained with UranyLess EM Stain 22 409 (Electron Microscopy Sciences, PA, USA) on a 200‐mesh carbon grid and examined. The size distribution and zeta potential were determined using NanoSAQLA (Otsuka Electronics) and ELSZ‐Neo (Otsuka Electronics).

### 2.2 In Vitro Study

#### 2.2.1 Cell Culture

The human epidermal keratinocyte cell line (HaCaT) was cultured in Dulbecco's Modified Eagle's medium supplemented with 10% fetal bovine serum (FBS; Biowest, USA) and 1.0% penicillin/streptomycin (penicillin 100 IU/mL, streptomycin). Cells were incubated in a humidified atmosphere containing 5.0% CO_2_ and 95% air at 37°C. The cells were subcultured when they reached 80%–90% confluency. Capsaicin (Sigma‐Aldrich) was used to activate TRPV1 and induce irritation in order to simulate skin sensitivity. HaCaT cells were treated with 50 μM capsaicin, the maximum nontoxic concentration, and subjected to enzyme‐linked immunosorbent assay (ELISA).

### 2.3 Cytotoxicity Assessment

Cytotoxicity was assessed using the water‐soluble tetrazolium salt (WST‐1) assay. WST‐1 assay solution (EZ‐CYTOX; DOGEN, Korea) was added at a medium volume of 10 wt% to human keratinocytes after culturing for 24 h. The cells were incubated for an additional 0.50 to 1.0 h at 37°C. The absorbance was measured at 450 nm using a microplate reader (SpectraMax i3x Multi‐Mode Detection Platform; Molecular Devices, USA). The reference absorbance was measured at 650 nm, and the results were corrected. The results are presented as the mean ± standard deviation of three independent experiments.

### 2.4 Evaluation of Cytokine Protein Expression Using ELISA

We incubated 1.0 × 10^5^ HaCaT cells per well in 24‐well plates for 24 h. Cells were treated with 0.5, 5.0, and 50 μg/mL of _L‐_glutamine, LA, and QLAsome or 1 μg/mL of capsazepine for 30 min, along with 50 μM capsaicin, and incubated for 24 h. Capsaicin and capsazepine were selected as TRPV‐1 activators and antagonists for capsaicin, respectively, for the expression of inflammatory mediators [21]. MCP1, IL1a, IL4, IL6, IL8, TNF‐a, MMP‐1, and MMP9 protein levels were quantified using an ELISA kit for humans (R&D Systems, Minneapolis, MN, USA). The results are presented as the mean ± standard deviation of three independent experiments.

### 2.5 Antibacterial Activity of QLAsome Against *S. aureus*

A single colony of *S. aureus* (ATCC 6538) was inoculated into 8.0 mL of tryptic soy broth medium (BD Diagnostics, Franklin Lakes, NJ, USA) and cultured for 24 h at 35°C to determine the minimum growth inhibitory concentration (MIC) against *S. aureus* . The prepared samples were dissolved in water or DMSO and diluted by a two‐fold serial titration. Subsequently, 0.10 mL of each diluted sample (experimental group) or solvent (control group) was added to 9.9 mL of the medium and 0.10 mL of the culture medium. The solutions were mixed thoroughly and incubated for 24 h at 35°C. Optical density (OD) was measured using a UV spectrophotometer after incubation. MIC was defined as the lowest concentration of an agent that achieved ≥ 90% growth inhibition. A single colony was inoculated into 8 mL of tryptic soy broth and incubated for 24 h at 35°C for the disk diffusion assay. Culture medium (0.10 mL) was spread on prehardened tryptic soy agar in a Petri dish with a sterilized paper disk at its center. The solution (0.020 mL) was dropped onto a paper disk at the center of a Petri dish and allowed to absorb. The paper disks were incubated for 24 h at 35°C. Growth‐inhibition zone diameter was measured after incubation to determine its width, which was calculated as follows:

$$W=\frac{T-D}{2}$$

T: Total diameter of the growth inhibition area (mm).

D: Diameter of the samples (mm).

W: Width of the growth inhibition area (mm).

### 2.6 Clinical Study Overview and Clinical Evaluation

A specialist clinical evaluation organization oversaw participant recruitment, evaluation, and all the clinical trials in this study. Clinical experiments were conducted in accordance with the tenets of the Declaration of Helsinki. This study adhered to the regulations on medicines, over‐the‐counter (OTC) drugs, cosmetics, and medical devices established by government agencies and the Bioethics and Safety Act. The trial was performed in accordance with the standard operating procedures, and all procedures were inspected and implemented by the quality assurance manager. Clinical evaluations for improvement in itchiness through barrier recovery and relief of skin sensitivity were conducted under the clinical management numbers KIDS‐BDH103‐SNC and KIDS‐BDH105‐SNC, respectively. The study period spanned from September 4, 2024, to October 22, 2024.

The inclusion criteria were as follows: (1) adult women aged 20–65 years with itchy skin; (2) transepidermal water loss (TEWL) ≥ 12 g/m^2^/h in the test area (3 cm below the cubital fossa, selected for its few sebaceous glands and thin skin barrier, making it sensitive to dryness and itching symptoms), as measured by Tewameter; (3) voluntary participation; and (4) absence of acute or chronic physical diseases, including skin diseases. The exclusion criteria were as follows: (1) total erythema, scaling, induration, and fissuring (ESIF) scale score ≥ 6; (2) administration of antibiotics, steroids, immunosuppressants, antihistamines, retinoids, or phototherapy related to skin diseases during the screening period; (3) skin barrier damage caused artificially by physical or chemical methods (e.g., peeling and use of peeling agents) in the test area during the screening period; and (4) sensitivity to cosmetics. The cream was manufactured and sent to the Clinical Evaluation Committee. Under the Ethics Committee's supervision, the evaluator explained the use of the product, precautions, and how to handle potential side effects to the participants and obtained their consent before proceeding with the clinical evaluation. The evaluation was performed in a double‐blind manner. The evaluation flow was as follows:

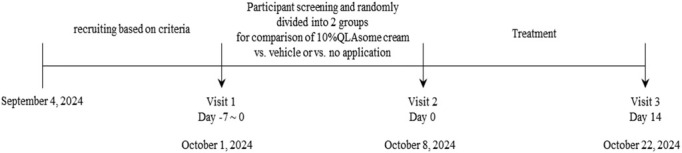

### 2.7 Itching Improvement in the Cubital Fossa Area via the Reduction of Inflammation and Recovery of Skin Barrier

A total of 24 Korean women, aged 22–63 years, were randomly divided into two groups. Twelve participants applied an even layer of ‘Round Lab Yak Kong panthenol ato‐cream with 10 wt% QLAsome’, based on the average percutaneous absorption rate of cosmetics and the viability limit of QLAsome in keratinocytes and fibroblasts (1.0 wt%, data not shown), to an area 3.0 cm below the cubital fossa for 2 weeks. An equivalent amount of the same formulation, without QLAsomes, was applied to the other side and was allowed to be absorbed. The remaining 12 participants applied the formulation on one side in the same manner, and no formulation was applied on the other side. The participants were prohibited from using any moisturizer or body scrub that could affect the test results, or from receiving treatments such as packs or massages.

### 2.8 Alleviating Facial Skin Sensitivity by Reducing Inflammation and Improving Skin Barrier

The face is the most exposed part of the body, making it highly susceptible to skin abnormalities. Fewer sebaceous glands were present in the cheeks. Consequently, it was selected as the measurement area because of its sensitivity for detecting dryness and skin barrier abnormalities. A total of 24 participants aged 22–63 years with skin sensitivity were selected using a lactic acid sting test and randomly divided into two groups for evaluation. The participants in the first group applied an even layer of 3Round Lab Yak Kong panthenol ato‐cream with 10 wt% QLAsome twice a day to one cheek for 2 weeks after washing their face. The vehicle was applied to the other cheek and allowed to be absorbed. The participants in the second group applied cream to one cheek twice daily for 2 weeks. No formulation was applied to the other cheek. Participants were instructed to avoid using any moisturizer or body scrub that could affect the test results and to avoid receiving treatments such as packs or massages.

### 2.9 Clinical Assessment

All evaluations were performed after stabilizing the area at constant temperature and humidity, followed by washing with the same cleanser. The temperature was maintained at 22°C ± 2.0°C and humidity at 50% ± 5.0% for 30 min to facilitate stabilization. The presence of skin abnormalities, such as erythema, edema, scaling, itching, stinging, burning, tightness, and prickling at the test site, was recorded. No adverse reactions were observed during evaluation.

### 2.10 Itching Assessment

Itching improvement was assessed using the visual analog scale (VAS) [22]. The average and maximum itching severity at 24 h before and after test substance application was evaluated using a 10‐cm VAS. The scores were categorized as follows: 0, no itching; 1 and 3, mild itching; 3–7, moderate itching; 7–9, severe itching; and 10, very severe itching.

### 2.11 Lactic Acid Sting Test

The initial screening was conducted using a self‐assessment questionnaire, followed by a lactic acid stinging test to identify individuals with skin sensitivity. Fifty microliters of 10 wt% lactic acid solution and 50 μL of 0.90% sodium chloride solution were applied to the nasolabial folds, and the stinging sensation (tingling) caused by each solution was evaluated 2.0 min after application using a four‐point scale. The scores were categorized as follows: 0, none; 1, mild; 2, moderate; and 3, severe. Skin sensitivity was deemed absent if no sensation was reported after 10 min, or if the score on the side with lactic acid application was the same or lower. Skin sensitivity was considered present if the score on the side with lactic acid application was significantly higher than that on the side with saline application (*p* < 0.05), as analyzed using the Wilcoxon signed‐rank test.

### 2.12 Transepidermal Water Loss

The Tewameter TM300 (Courage + Khazaka Electronic GmbH, Cologne, Germany) was used to apply the same amount of pressure to an area 3.0 cm below the elbows or faces of the test participants. TEWL was continuously measured until a stable value was obtained, with the average of the last three values used in the analysis.

### 2.13 SC Hydration

The capacitance, an indicator of SC hydration in the test area, was measured using a Corneometer CM 825 (Courage + Khazaka Electronic GmbH, Cologne, Germany). The average of the last six values was used in the analysis.

### 2.14 Skin Erythema

The area 3.0 cm below the cubital fossa of both arms was evaluated using a spectrophotometer (CM‐2600D; Konica Minolta, Tokyo, Japan). The evaluation was performed three times consecutively using the same tester at the same pressure. The average measurement result was determined and used in the analysis. The measured values of the spectrophotometer were comprised of three factors: L*, a*, and b. The a* value, which indicates erythema, was used for the analysis. The full‐eye imaging system VISIA‐CR (VISIA Clinical Research, Canfield Scientific Inc., USA) and an image analysis program (ImageJ, National Institutes of Health, USA) were used to evaluate the face. Both sides of the face (test and control substance application areas) were evaluated and the a* value was used in the analysis.

### 2.15 Visual Assessment Using ESIF

A dermatologist performed a visual evaluation using the ESIF scale to evaluate the improvement in itching and alleviation of skin sensitivity through the recovery of skin barrier function following the application of the test substance. This evaluation was conducted 2 weeks before and after the test substance was administered. The total score, obtained by adding the four variables erythema, scale, duration, and fissures, ranged from 0 to 12. A score of > 6 indicates the requirement for dermatological treatment. Patients with a score > 6 were excluded from the study.

### 2.16 Sensory Evaluation

The satisfaction level of participants following the use of the test substance was evaluated using a satisfaction index. The scores were categorized as follows: 1 (very good), significant improvement in itching; 2 (good), overall improvement in itching; 3 (unchanged), no difference from before application; 4 (worse), increase in itching; and 5 (very worse), significant increase in itching.

### 2.17 Statistical Analysis

All statistical analyses were conducted using SPSS 17.0 for the Windows program. The Shapiro–Wilk test was used to test the normality of the measured values before and after applying the test substance. A paired t‐test analysis was performed when normality was satisfied, and Wilcoxon signed‐rank test analysis was performed when normality was not satisfied. When comparing the test and placebo area or application area and the non‐application area, the Shapiro–Wilk test was used to test for normality of the measurements before and after use. If normality was satisfied, an independent t test was used. If normality was not satisfied, post hoc analysis was performed using the Wilcoxon signed‐rank test after analysis using the Friedman test, a non‐parametric statistical method.

## 3 Results

### 3.1 Preparation and Morphology of QLAsome

**FIGURE 1:**
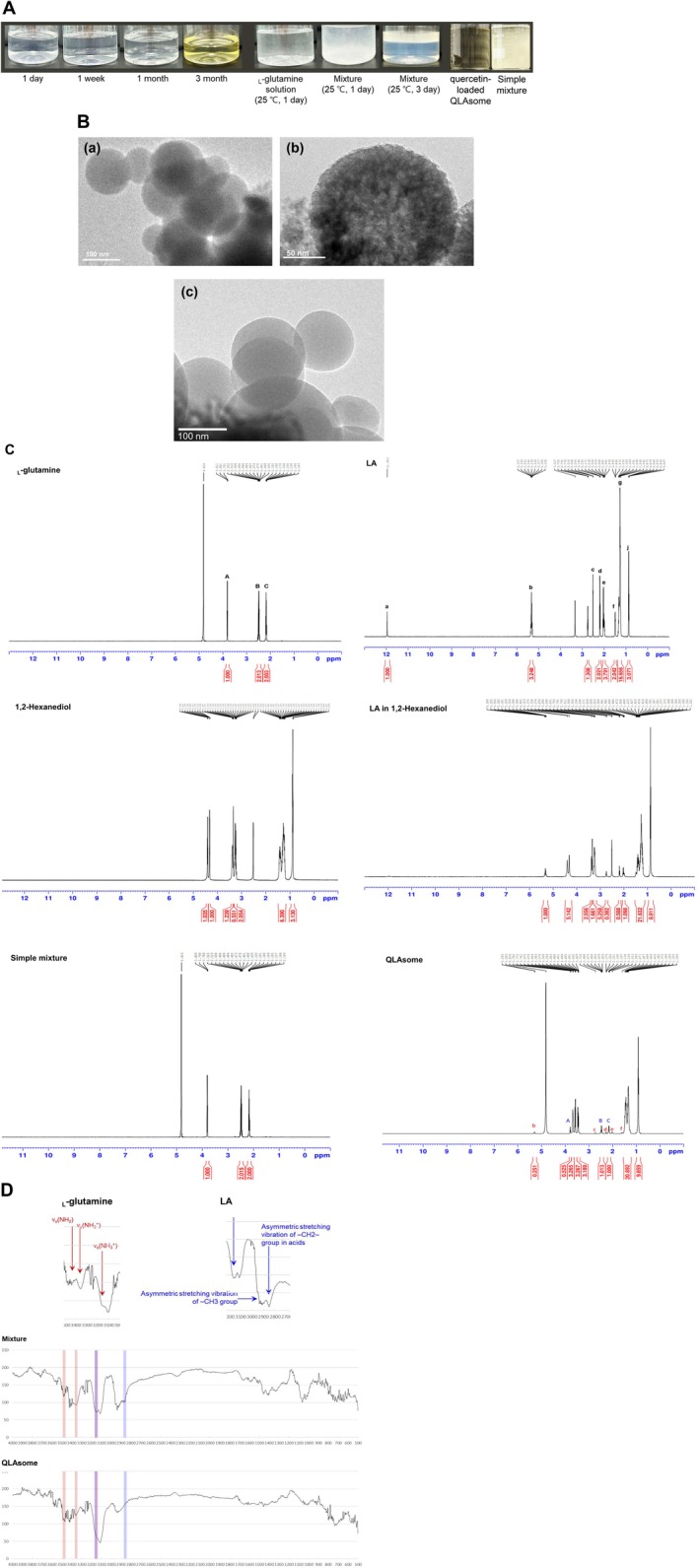
(A) Long‐term stability of the QLAsomes solution compared to that of the simple mixture and _L‐_glutamine aqueous solution manufactured with the same content. Macroscopic stability of the QLAsomes (B) Transmission electron microscopy (TEM) images of QLAsomes (a, b) and quercetin‐loaded QLAsomes (c). (C) Results of 1H‐NMR analyses of _L‐_glutamine, linoleic acid, 1,2‐hexanediol, a simple mixture with the same contents, and QLAsomes. (D) Results of the IR spectroscopic analysis of the simple mixture and QLAsomes were compared with those of the amine group of _L‐_glutamine and the carboxylic group of linoleic acid.

According to the American Chemical Society, the solubility of _L‐_glutamine in water is 25 g/L (20°C); however, its solubility in water increases to > 50 g/L (20°C) following the creation of ion pairs with LA. The aqueous solution of glutamine and the simple physical mixture, prepared with the same composition as QLAsomes, exhibited precipitation or phase separation within 1–3 days (Figure 1). However, QLAsomes remained stable for more than 3 months. The pH value of QLAsomes was maintained at 4.6 throughout the macroscopic stability evaluation. TEM revealed that QLAsome formed spherical nanovesicles (Figure 1B (a), (b)) with an average diameter of 164.6 ± 3.1 nm and a surface charge of −4.6 mV measured by zeta potential (Table 1). Quercetin‐loaded QLAsomes were formulated as a proof‐of‐concept experiment on nanovesicles to deliver antioxidants that are unstable or poorly soluble in water, and their morphological characteristics were observed. Table 1 presents the particle size, polydispersity index of the size distribution, and zeta potential of the vesicle formulations. The intensity‐average vesicle size and zeta potential were 213.2 ± 1.5 nm and −6.0 mV, respectively. No significant morphological changes were observed in the TEM images (Figure 1B (c)). Quercetin was stably loaded (0.010 wt %) and precipitated at concentrations of 0.025 wt% or higher under the conditions used in this study. Evaluation of macroscopic stability for > 3 months revealed no changes in quercetin‐loaded QLAsomes (data not shown).

**TABLE 1:** Vesicular characteristics of glutamine linoleate vesicles (QLAsomes) or quercetin‐loaded QLAsome dispersions.

Sample	Size (nm)	PDI	Zeta potential (mV)
QLAsome	164.6 ± 3.1	0.116	−4.56
Quercetin loaded QLAsome	213.2 ± 1.5	0.250	−6.02

### 3.2 Binding Characteristics Between _L‐_Glutamine and LA Using Spectroscopic Analysis

The structural differences between _L‐_glutamine and LA were identified using NMR. The chemical shifts identified in the 1H‐NMR spectra reflect the state of the nuclear environment and provide information on the molecular interactions of QLAsomes in aqueous solutions (Figure 1C). A comparison of the individual spectra of LA and 1,2‐hexanediol with those of a solution in which LA was dispersed in 1,2‐hexanediol revealed no chemical shifts. Similarly, a comparison between simple mixtures of _L‐_glutamine and LA (dispersed in 1,2‐hexanediol) with the same composition as QLAsomes revealed no electronic chemical shifts. These results indicated that phase separation occurred without vesicle formation when the components were mixed with the same composition. However, during QLAsome formation, the “a” electron peak in LA disappeared owing to proton dissociation from the carboxyl group and hydrogen bond formation with _L‐_glutamine. The “d, f, and e” peaks exhibited chemical shifts of 0.28, 0.12, and 0.032 ppm, respectively, indicating that interbond influence was greater when proton was near the carboxyl group. Peak “A,” corresponding to a proton near the backbone amino acid of _L‐_glutamine, shifted by 0.14 ppm due to electron transfer by hydrogen bonding after QLAsome formation. These 1H‐NMR results are consistent with those presented in Figure 1D, which analyzes the bonds between functional groups using FT‐IR. The positions of the amine group of _L‐_glutamine (indicated in red) and the carboxyl group of LA (indicated in blue) were verified in The IR spectra of the individual components, as described previously [23, 24]. The IR spectrum of the sample mixture was the sum of each peak; however, the spectrum of QLAsomes exhibited conformational changes in the two functional groups (indicated in red and blue) due to strong hydrogen bonding.

### 3.3 Effect of QLAsome on the Suppression of Capsaicin‐Induced Skin Sensitivity Factors in Keratinocytes

**FIGURE 2:**
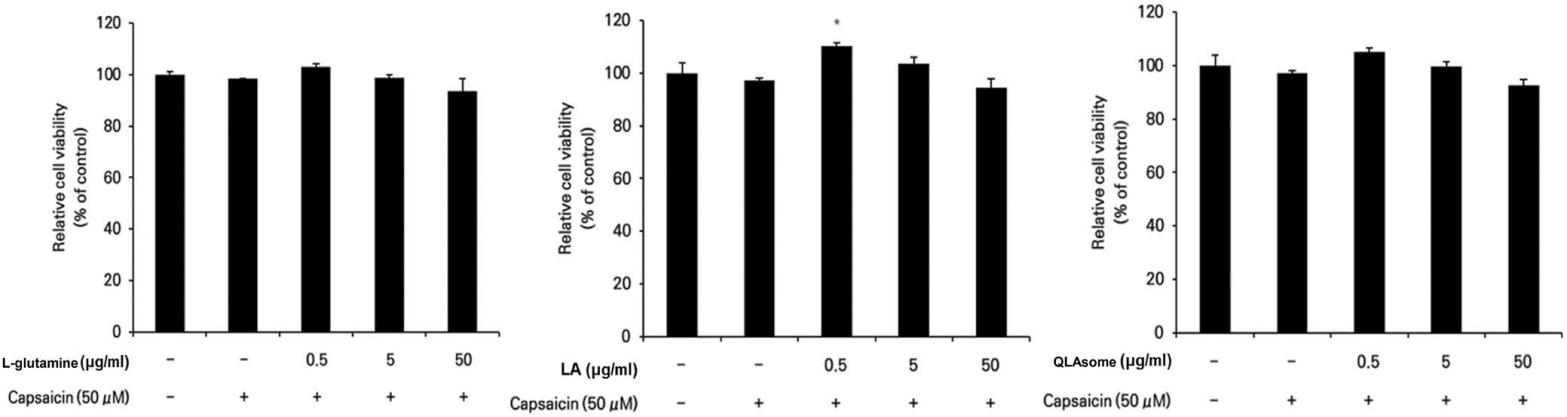
Cytotoxicity assessment of keratinocytes treated with _L‐_glutamine, linoleic acid, or QLAsomes with capsaicin.

**FIGURE 3:**
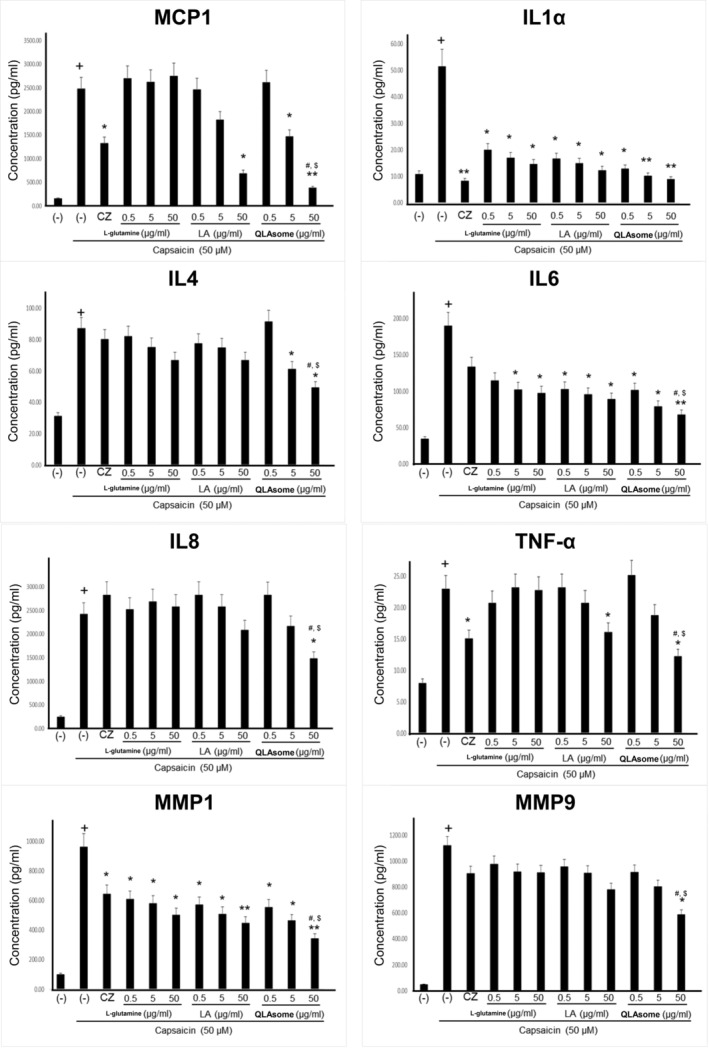
Inhibitory effects examination of _L‐_glutamine, linoleic acid, and QLAsome after TRPV1 activation by capsaicin in HaCaT cells on the expression of CCL2/JE/MCP1, IL1α/IL1F1, IL4, IL6, IL8/CXCL8, TNF‐α, MMP1, and MMP9. Each column presents the mean ± SD. † indicates a significant difference before and after capsaicin treatment at *p* < 0.05. * or ** indicates a significant difference between the presence or absence of agent treatment under capsaicin treatment conditions, # denotes a significant difference between QLAsome and _L‐_glutamine, and $ represents a significant difference between QLAsome and LA. One or two symbols represent *p* < 0.05 or *p* < 0.01, respectively.

The effects of QLAsomes on the expression of eight inflammatory cytokines in capsaicin‐induced TRPV1 activating HaCaT cells were investigated in the present study. The cytokines evaluated in this study were related to the initiation of inflammatory responses and pruritus induction. IL4 is a key cytokine involved in the aggravation of AD lesions by mediating immune responses, sustaining inflammation, and causing abnormalities in skin barrier function. MMP‐1 and MMP‐9 degrade collagen and gelatin, respectively, thereby inducing deterioration of the skin barrier. Keratinocytes treated with _L‐_glutamine, LA, or QLAsomes, followed by the addition of capsaicin, did not exhibit cytotoxicity at any of the evaluated concentrations (Figure 2). Cytokine expression levels were determined using ELISA based on the amount of protein released by each factor. The cells were treated with various concentrations of _L‐_glutamine, LA, QLAsome, or 1.0 μg/mL of capsazepine for 30 min, and 50 μM capsaicin was added to the culture medium and incubated for 24 h. The protein level of each factor secreted into the medium was measured. Notably, TRPV1 activation by capsaicin increased the protein levels of cytokines by 3–25‐fold. _L‐_glutamine exhibited statistically remarkable inhibitory effects on IL1a, IL6, and MMP‐1 at the tested concentrations (*p* < 0.05) (Figure 3). Similarly, LA exhibited statistically significant inhibitory effects on IL1a, IL6, and MMP‐1 at all treatment concentrations (*p* < 0.05), CCL2/JE/MCP1 CCL2/JE/MCP1 and TNF‐α only at 50 μg/mL (p < 0.05). In contrast, QLAsome statistically remarkably inhibited the secretion of all eight cytokines, and the levels were similar to or higher than those in cells treated with capsazepine (IL4, IL8/CXCL8, TNF‐α, MMP‐9: *p* < 0.05; CCL2/JE/MCP1, IL1a, IL6, MMP‐1: *p* < 0.01) (Figure 3). In particular, as a factor that is significantly associated with barrier abnormalities and skin sensitivity, the cytokine IL4 was significantly downregulated by more than 26% compared to the individual components, and the expression of MMP1 showed a significant decrease of 32% and 23% compared to _L‐_glutamine and LA, respectively (IL4 and MMP1: *p* = 0.02 and 0.03, respectively).

### 3.4 Growth‐Inhibitory Effect of QLAsomes on *S. aureus*

**FIGURE 4:**
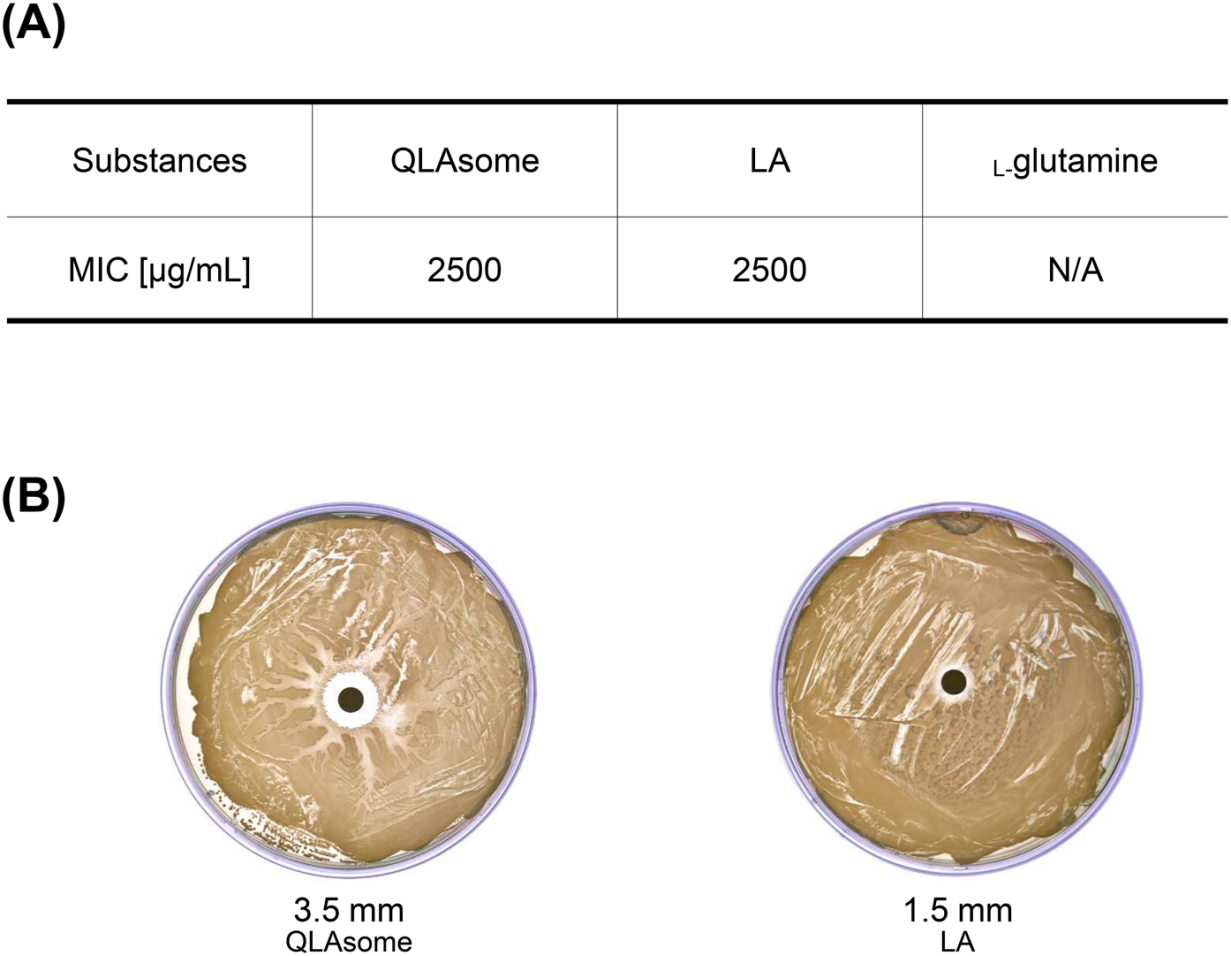
(A) Minimum inhibitory concentrations (MIC) against *S. aureus* bacteria (μg/mL). (B) Results of the disk diffusion assay after treatment with QLAsomes or linoleic acid.

Figure 4A shows the growth inhibitory effects of LA dispersed in 1,2‐hexanediol, _L‐_glutamine solution, and QLAsomes on *S. aureus* . MIC evaluation revealed that _L‐_glutamine, a component of QLAsomes, had no antibacterial activity against *S. aureus* . The MIC revealed that LA dispersed in 1,2‐hexanediol with the same content as QLAsome was formed at 2500 μg/mL. The MICs of the representative ‘non‐antibiotic’ antibacterial agents, salicylic acid and benzoyl peroxide, were 4000–8000 μg/mL and 2048 μg/mL (75% benzoyl peroxide), respectively [25]. The evaluation results revealed that QLAsome exhibited efficacy similar to that of well‐known non‐antibiotic antibacterial agents. The diffusion assay indicated that QLAsomes may be more effective in inhibiting *S. aureus* than LA, owing to their ability to form a wider growth inhibition zone (Figure 4B). Despite the lack of _L‐_glutamine antibacterial activity, the structural change resulting from the intermolecular bond between _L‐_glutamine and LA indicated that the enhanced antibacterial activity may result from a higher diffusion efficiency in the media. Therefore, it might be effective in preventing skin sensitivity and related diseases caused by *S. aureus* .

### 3.5 Clinical Evaluation on Itching

**FIGURE 5:**
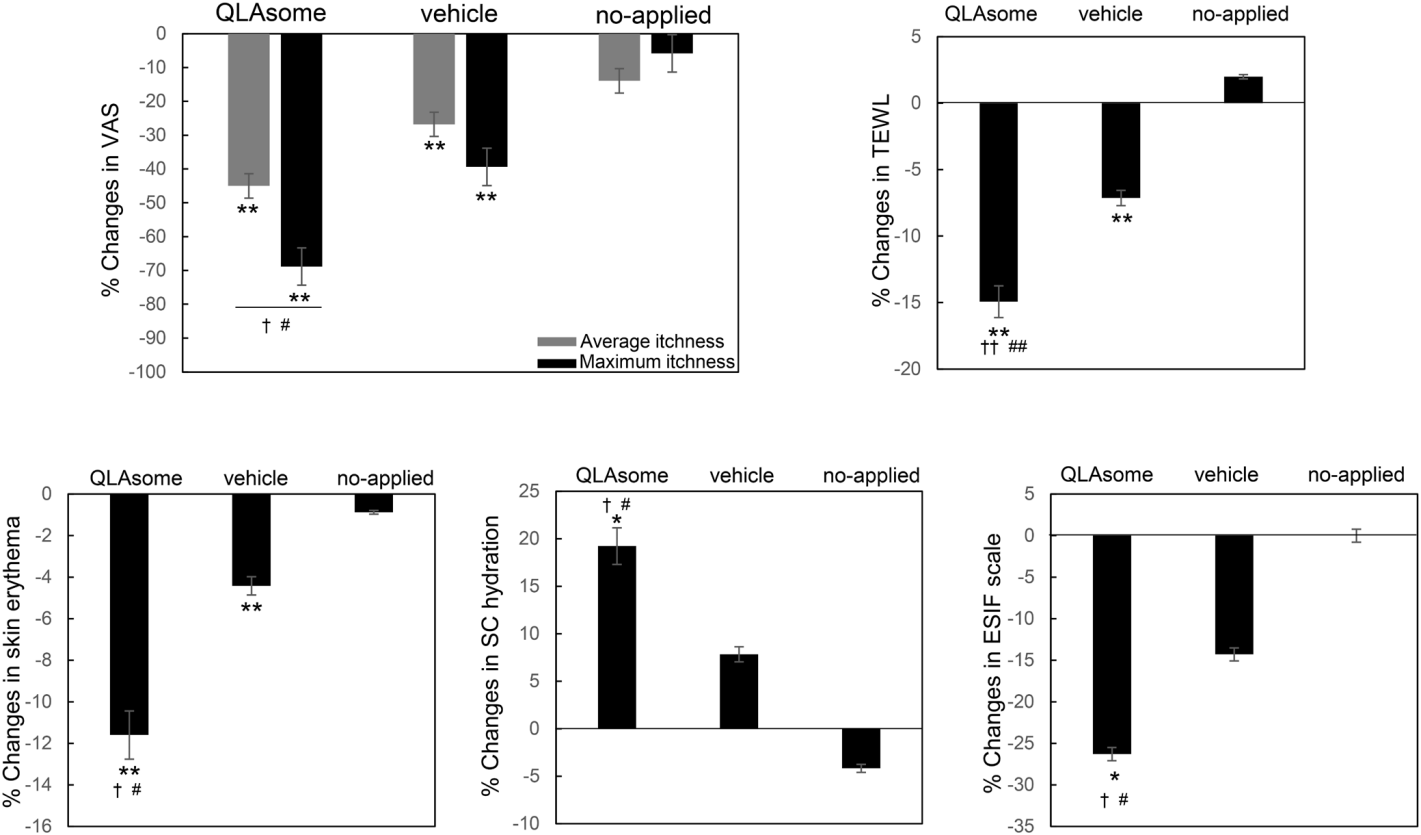
Changes in itchiness (changes in mean and maximum itchiness assessed by VAS), TEWL, skin erythema, SC hydration, and ESIF scale scores on the lower part of the arm in groups treated with QLAsome‐containing cream, vehicle, and no cream for 2 weeks. (* indicates a significant difference before and after application, # represents a significant difference between test and placebo, and † indicates a significant difference between test and no application. One or two symbols represent *p* < 0.05 or *p* < 0.01, respectively).

Participants with sensitive skin due to a weakened skin barrier were selected based on the criteria mentioned in the Methods section. A clinical study was conducted to determine the effects of erythema and improvement of the skin barrier on itching and sensitive skin. The degree of relief from pruritus, recovery of the skin barrier, and skin erythema were evaluated following the application of a cream containing QLAsomes to a 3‐cm area below the cubital fossa of both arms of participants with pruritus. In addition, subjective and instrumental evaluations of the alleviation of skin sensitivity were performed on the faces of the participants with skin sensitivity selected using the lactic acid stinging test. In all the clinical trials conducted, no participants withdrew from the clinical evaluation owing to irritation symptoms or side effects. Figure 5 shows that the average improvement rates after 2 weeks were 45% and 27% for the side where the QLAsome cream was applied and the side where the placebo was applied, respectively, compared with those before use (QLAsome *p* = 0.002, placebo *p* = 0.003). In particular, the maximum itching improvement rate was 69% when QLAsomes cream was applied (*p* = 0.002), and a statistically significant improvement was confirmed in the comparative evaluation of all groups (*p* = 0.021). Through the improvement of TEWL, skin erythema, and SC hydration, QLAsomes contributed to skin barrier improvement, which in turn led to perceived relief of itching. Specifically, TEWL improved by 15% (*p* = 0.002) and skin erythema improved by 12% (*p* = 0.002). The SC hydration results showed the same trend, and in the comparison between groups, QLAsomes showed a significantly greater improvement than all other groups (*p* = 0.002).

### 3.6 Clinical Evaluation on Skin Sensitivity

**FIGURE 6:**
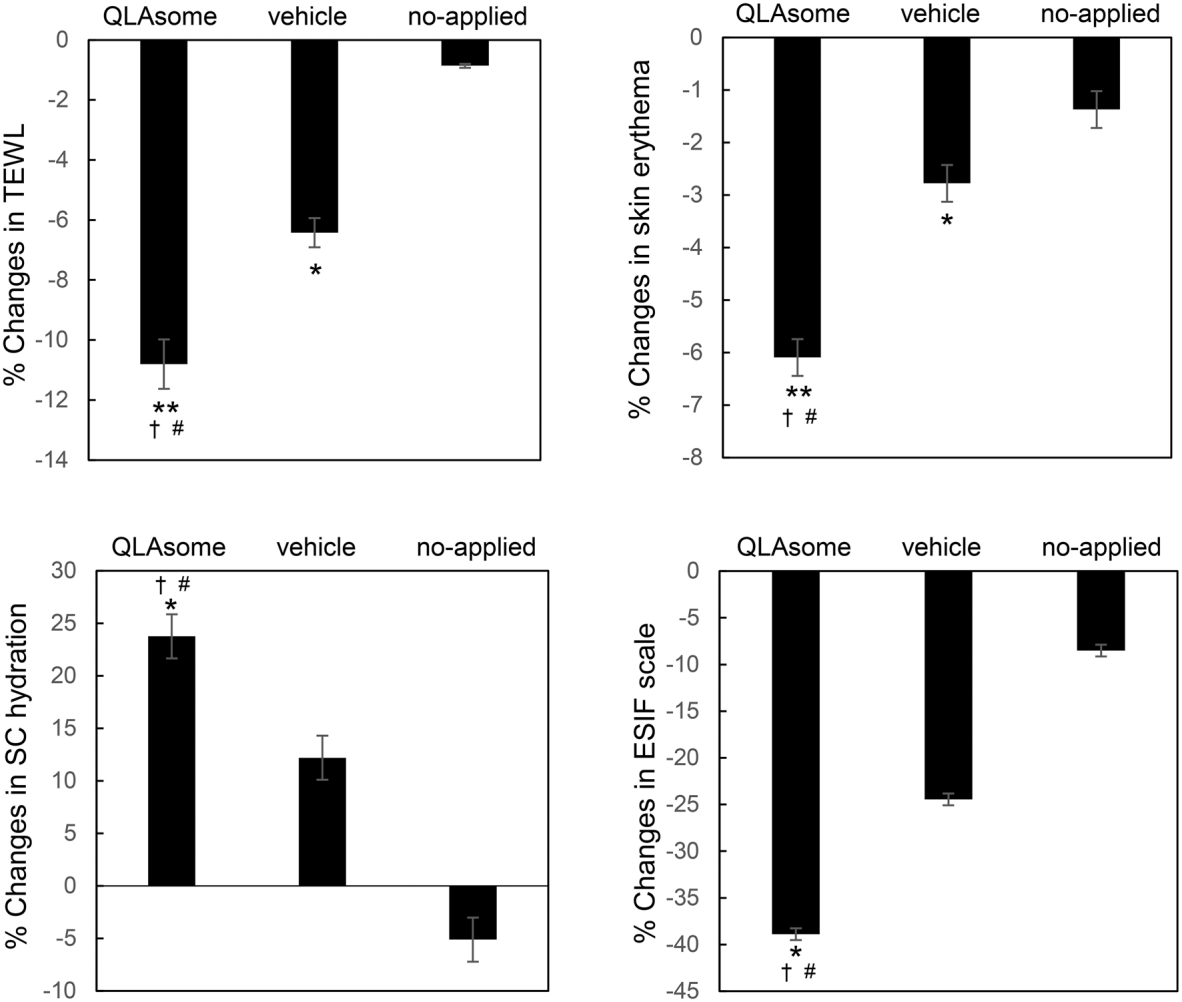
Changes in TEWL, skin erythema, SC hydration, and ESIF scale in the cheek in groups that received a cream containing QLAsomes, vehicle, and no cream for 2 weeks (* indicates a significant difference before and after application, # represents a significant difference between test and placebo, † indicates a significant difference between test and no application. One or two symbols represent *p* < 0.05 or *p* < 0.01, respectively).

Figure 6 shows the changes in TEWL, skin erythema, and SC hydration on the faces of the participants with skin sensitivity selected by the lactic acid challenge test over 2 weeks. Although the evaluated areas and symptoms differed, the improvement effects of QLAsomes on chronic inflammation and skin sensitivity showed a similar pattern. Application of QLAsome cream significantly improved TEWL, erythema, and SC hydration over 2 weeks compared to placebo (comparison between groups *p* = 0.011). Specifically, QLAsome cream application improved TEWL by 10%, erythema by 6.1%, and SC hydration by 24% (TEWL *p* = 0.005, erythema, *p* = 0.007; SC hydration, *p* = 0.02).

### 3.7 Expert Visual Evaluation and Sensory Evaluation

These evaluations revealed a consistent pattern of improvement in itchiness and skin sensitivity, as reflected in both the satisfaction questionnaire and the expert visual evaluation using the ESIF scale. A visual assessment conducted by the dermatologist using the ESIF scale revealed improvements of 26% and 39% in the cubital fossa and cheek, respectively, following the application of the QLAsome cream (Figures 5 and 6). A statistically significant improvement in both evaluations was observed following the application of the QLAsome cream (*p* < 0.05). In contrast, the application of the placebo yielded improvements of only 14% and 24% in the cubital fossa and cheek, respectively, and non‐application resulted in almost no change in the scale. The intergroup comparison revealed a statistically significant improvement in the findings of both evaluations in the QLAsome cream group compared to those in the vehicle and no‐application groups (*p* < 0.05). The questionnaire assessing patient satisfaction after using the cream on the lower arm or face for 2 weeks revealed a significant difference in satisfaction with the QLAsome cream group (Tables 2 and 3). Particularly, in the evaluation of itching improvement, a statistically significant difference (*p* < 0.01) was observed between the test and placebo or application, and between application and no application.

**TABLE 2:** Questionnaire‐based evaluation of satisfaction with itching improvement after 2 weeks of use.

Question	QLsome cream		Vehicles		No applied	
1 Very good (Itching was significantly improved overall)	7	Average** 1.42^a^	0	Average 3.25	0	Average 3.42
2 Good (Itching was improved)	5		2		0	
3 Unchanged (No difference from before use)	0		5		7	
4 Worsening (Worsening itching overall)	0		5		5	
5 Very worse (Itching is significantly worse overall)	0		0		0	

** Significant difference between the vehicle application group at *p* < 0.01.

^a^ Significant difference between the no‐application group at *p* < 0.01.

**TABLE 3:** Questionnaire‐based evaluation of satisfaction with facial skin sensitivity alleviation after 2 weeks of use.

Question	QLsome cream		Vehicles		No applied	
1 Very good (erythema was significantly improved overall)	8	Average* 1.20^a^	2	Average 1.80	0	Average 3.00
2 Good (erythema was improved)	2		8		0	
3 Unchanged (no difference from before use)	0		0		10	
4 Worsening (worsening erythema overall)	0		0		0	
5 Very worse (erythema is significantly worse overall)	0		0		0	

* Significant difference between the vehicle application group at *p* < 0.05.

^a^ Significant difference between the no‐application group at *p* < 0.01.

## 4 Discussion

In the present study, QLAsomes, a vesicle comprising an ion pair of _L‐_glutamine and LA, were manufactured as a cosmetic agent to alleviate irritation associated with skin sensitivity. Furthermore, the morphological characteristics and macroscopic stability of cosmetic ingredients were evaluated. The changes in the expression of proinflammatory cytokines (MCP1, IL1α, IL4, IL6, IL8, and TNFα) and MMPs (MMP1 and MMP9) in HaCaT keratinocytes, wherein TRPV1 was activated by capsaicin, were evaluated to verify the in vitro efficacy of QLAsome as a cosmetic agent. The expression of all the evaluated proteins was significantly suppressed. These downregulated cytokines and proteases are significantly associated with inflammatory response initiation, inflammation through external infection, and the onset or worsening of dermatitis symptoms. These in vitro results indicated that QLAsomes can improve skin sensitivity by inhibiting the expression of cytokines that cause skin irritation. The criteria described in previous studies were used to select participants and establish methods for evaluating the alleviation of skin sensitivity following the use of QLAsomes as cosmetic agents. Specifically, TEWL was the main criterion for selecting sensitized skins. The average TEWL values in the lower part of the cubital fossa were 8.3 ± 2.2 g/m^2^/h and 8.9 ± 2.4 g/m^2^/h among healthy Korean men and women, respectively [26]. The average seasonal differences at the same site in summer and winter among Korean men were 10 ± 2.9 g/m^2^/h and 8.2 ± 2.5 g/m^2^/h, respectively [27]. Thus, in the present study, a TEWL of ≥ 12 g/m^2^/h was set as the standard for an abnormal skin barrier. This standard was deemed appropriate considering that the evaluation period was October. The participants included in the evaluation of skin sensitivity relief were selected objectively using the lactic acid stinging test. Itching was assessed subjectively. VAS was used to numerically evaluate itching, as described previously [28]. The dermatologist conducted a visual evaluation using the ESIF scale to account for differences in satisfaction, which is a subjective measure. ESIF evaluates and scores four elements: erythema, scaling, induration, and fissuring. Participants with ESIF scale scores of ≥ 6 points prior to evaluation were excluded, and changes in the VAS and ESIF scales were verified. The effects of QLAsomes were assessed in 24 participants selected using these criteria. Although the evaluation scale was small at the experimental model level, the intergroup comparison revealed a significant difference (*p* < 0.05). A comparison of improvements in itching and skin sensitivity between the groups revealed that the application of the cream containing QLAsomes alleviated itching in the cubital fossa of both arms. Furthermore, QLAsomes alleviated skin sensitivity on the face as well as TEWL, SC hydration, and skin erythema. These findings suggest that QLAsomes alleviate skin sensitivity by promoting skin barrier recovery and suppressing inflammation. The difference in improvement rates between the groups was more pronounced when evaluating the cubital fossa of both arms, a relatively thin and sensitive area compared to the face. This numerical difference was particularly large for subjective satisfaction reported by the participants.

Unlike other cosmetic formulations (e.g., skin toners or emulsions), creams are more effective at improving TEWL because of their relatively high viscosity and polyol and oil content. These characteristics led to an improvement in the vehicle alone in just 2 weeks. However, the significant improvements in TEWL, SC hydration, and skin erythema following QLAsome use, as well as the high level of perceived efficacy reported by the participants, suggest that QLAsomes may be a suitable cosmetic ingredient for skin sensitivity. Compared to previous studies, cytokine expression inhibition by capsaicin showed a superior effect on IL6, IL8, and MMP9. Additionally, QLAsomes demonstrated a stronger inhibitory effect on IL1a, IL6, and TNFα than a previous study evaluating paeonol/madecassoside in a nanoemulsion [29, 30]. Skin sensitization occurs due to various factors, and the inhibitory effect on inflammatory cytokines alone cannot fully explain the clinical efficacy of QLAsomes. However, this comparison suggests that QLAsomes could be used as an effective agent. A comparison between Round Lab Yak Kong panthenol ato‐cream with QLAsome and formulations used in previous clinical studies revealed effects equivalent to or greater than those of Aquammunist + Calming Complex ( *Annona cherimola* Fruit Extract + Niacinamide), Atopalm Multi‐Lamellar Emulsion Cream, and Physiogel Intensive Cream in terms of barrier recovery through TEWL improvement [31, 32].

The QLAsome unit, a water‐soluble ion pair of amino acids and fatty acids, is superior to previous commercial soothing systems because it can be easily applied to toners and low‐viscosity emulsions. However, because hydrogen bonding—the driving force of the ion pair—can be affected by the surrounding components, a solubilizer may be needed for long‐term formulation stability. From a green chemistry perspective, QLAsomes have the advantage of reducing the use of chemically synthesized surfactants. For the evaluation scale, there may have been a potential bias, as the evaluation was conducted with 24 participants. To minimize such errors, we selected participants based on previous studies, conducted the evaluation in a double‐blind manner, and eliminated factors that affected the skin other than the evaluated product to minimize external confounding factors. The size of the participant population in the existing study ranged from 24 to 60 participants, and a comparison of the results of the present study with those of the existing studies mentioned above is considered reasonable.

This study aimed to clinically evaluate the relief of skin sensitivity based on objective evidence. However, VAS has limitations in objectifying subjective itchiness. Additional evaluations are required to reduce potential bias. Furthermore, a comparative study assessing the long‐term stability of QLAsomes, changes in perceived effects, and subjective satisfaction compared with existing sensitive creams could provide more comprehensive insights. Since QLAsomes function as delivery carriers that can contain other agents, they would show higher utility value if further comparative evaluations were conducted with other delivery systems such as liposomes, niosomes, or microemulsions.

## 5 Conclusion

In this study, we demonstrated that the simultaneous application of _L‐_glutamine and LA through QLAsome synthesis was effective in sensitive skin. QLAsomes effectively suppressed the expression of inflammatory cytokines, MMPs, and the growth of bacteria and clinically indicated a recovery effect of the skin barrier. Clinical evaluation of QLAsomes at various concentrations for easier application in diverse formulations, as well as additional testing on facial areas such as cheeks, will enhance its appeal as a versatile ingredient. QLAsomes showed greater improvement in skin sensitivity than the individual effects of the ingredients, and it is significant that nanovesicles, previously used only as carriers, now provide a soothing effect. With their ability to encapsulate drugs, QLAsomes have the potential to be widely used as effective skin‐soothing ingredients in the pharmaceutical and cosmetic industries. In addition to soothing sensitive skin, it could enable continuous skincare application with less irritation through step‐by‐step application of retinoids and chemical exfoliators.

## Author Contributions

All authors performed the research. Koo Chul Kwon: designed the research study, analyzed the data, and wrote the manuscript. All authors have read and approved the final manuscript.

## Conflicts of Interest

The authors declare no conflicts of interest.

## Data Availability

The data that support the findings of this study are available from the corresponding author upon reasonable request.
